# Gender-Related Discrepancies in Short-Term Outcomes in Patients Undergoing Off-Pump Coronary Artery Bypass Grafting Surgery

**DOI:** 10.3390/jcm12062202

**Published:** 2023-03-12

**Authors:** Ihor Krasivskyi, Ilija Djordjevic, Borko Ivanov, Kaveh Eghbalzadeh, Clara Großmann, Stefan Reichert, Medhat Radwan, Rodrigo Sandoval Boburg, Anton Sabashnikov, Christian Schlensak, Thorsten Wahlers, Christian Jörg Rustenbach

**Affiliations:** 1 Department of Cardiothoracic Surgery, University Hospital Cologne, 50937 Cologne, Germany; 2 Department of Cardiothoracic Surgery, Helios Hospital Siegburg, 53721 Siegburg, Germany; 3 Department of Cardiothoracic Surgery, University Hospital Tuebingen, 72016 Tuebingen, Germany

**Keywords:** sex, coronary artery disease, OPCAB, mortality

## Abstract

The sex differences in patients undergoing off-pump coronary artery bypass grafting (OPCAB) surgery are still unclear. Our aim was to investigate the impact of gender on short-term outcomes in males and females after off-pump bypass procedures. Our research was designed as a double-center retrospective analysis. Generally, 343 patients (men (n = 255) and women (n = 88)) who underwent an OPCAB procedure were included in our study. To provide a statistical analysis of unequal cohorts, we created a propensity score-based matching (PSM) analysis (men, n = 61; women, n = 61). The primary endpoint was all-cause in-hospital mortality. Dialysis, transient ischemic attack (TIA), low cardiac output syndrome (LCOS), reoperation due to postoperative bleeding, wound infection and duration of hospital stay were secondary outcomes in our analysis. No significant differences were detected within the male and female groups regarding age (*p* = 0.116), BMI (*p* = 0.221), diabetes (*p* = 0.853), cardiogenic shock (0.256), STEMI (*p* = 0.283), NSTEMI (*p* = 0.555) and dialysis (*p* = 0.496). Males underwent significantly more frequently (*p* = 0.005) total-arterial revascularization with T-graft technique (*p* = 0.005) than females. In contrast, temporary pacer use was significantly higher (*p* = 0.022) in females compared to males. The in-hospital mortality rate was not significantly higher (*p* = 0.496) in the female group compared to the male group. Likewise, secondary outcomes did not differ significantly between the non-adjusted and the adjusted groups. Based on our findings, gender has no impact on short-term outcomes after OPCAB surgery.

## 1. Introduction

Cardiovascular diseases, including coronary artery disease, are associated with the highest mortality rate in western Europe [1]. Despite prophylactic and therapeutic efforts in the last decades, the prevalence of this disease continues to grow [2]. On-pump and/ or off-pump coronary artery bypass grafting surgery is a common and effective procedure used to treat triple vessel disease [1,2]. The discrepancies between both procedures regarding morbidity and mortality have been critically discussed in the last decades [3,4,5].

Woman generally present a higher prevalence of cardiovascular risk factors, including arterial hypertension, diabetes, dyslipidemia, and peripheral vascular disease (PVD), compared to men [6,7,8]. This could be explained by the fact that females develop coronary artery disease much later than males due to the protective role of sex hormones [9]. In addition, a smaller coronary artery diameter in women compared to men could influence the higher mortality rate after cardiac surgery [10,11]. Moreover, various studies have shown that coronary hyperreactivity, microvascular dysfunctions, plaque erosions, and distal microembolizations are more common among women compared to men [12,13]. A lower use of arterial grafts in females has also been associated with adverse outcomes and could affect survival [13]. Likewise, several studies have shown a significantly higher morbidity rate in the female group compared to the male group after bypass surgery [14,15]. Female gender was also found to be an independent predictor of higher mortality after a bypass procedure [16]. In contrast, other authors have mentioned that female gender was not associated with adverse outcomes and higher mortality after a CABG procedure [17,18]. However, the majority of the above-mentioned trials referred to patients who underwent both on-pump and off-pump CABG procedures [14,15,16,17,18].

Differences in gender outcomes in patients undergoing only OPCAB surgery are controversial [9,17,19]. Fu et al. [9] mentioned that early mortality did not differ significantly between both sex groups after OPCAB procedure. Likewise, further authors analyzed 776 patients who underwent an OPCAB procedure and showed no difference regarding mortality between males and females postoperatively [20]. In contrast, Puskas et al. [21] found that off-pump bypass surgery was associated with significantly lower (*p* = 0.001) mortality in women compared to men. 

Consequently, our aim was to evaluate whether the OPCAB technique could impact early outcomes in both sex groups. Thus, we investigated gender-related discrepancies in short-term outcomes in patients who underwent the OPCAB procedure in our heart centers.

## 2. Materials and Methods

**Figure 1:**
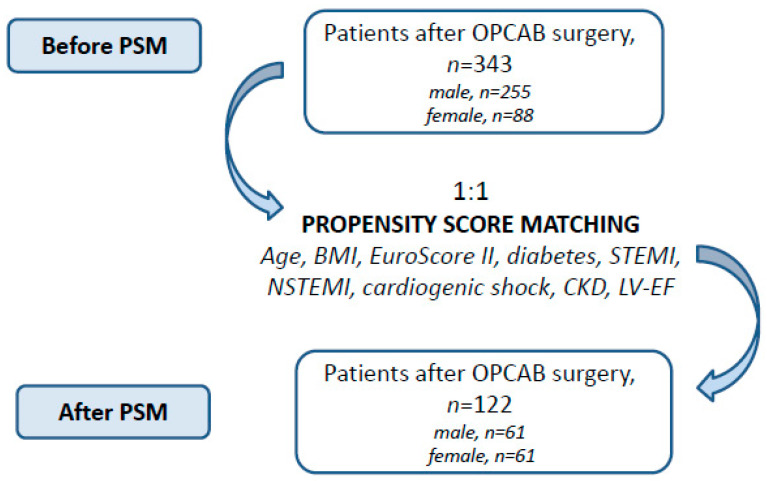
Study groups after OPCAB surgery before and after PSM. OPCAB: off-pump coronary artery bypass grafting. PSM: propensity score matching. CKD: chronic kidney disease. BMI: body mass index. STEMI: ST-elevation myocardial infarction. STEMI: non-ST-elevation myocardial infarction. LV-EF: left ventricular ejection fraction.

Our study was a retrospective analysis of a double center-retrospective OPCAB cohort, which included 343 patients who underwent off-pump coronary artery bypass procedure for coronary artery disease from January 2017 until November 2022 in the University hospitals in Cologne and Tuebingen. We formed two groups to investigate potential sex-related differences in early clinical outcomes: men (n = 255) and women (n = 88). To account for the unequal cohort sizes, we performed a propensity score-based matching (PSM) analysis using the methods previously described [22]. The details of the analysis are provided in Figure 1.

### 2.1. Surgical Procedure

All surgical procedures were performed using the OPCAB technique. Patients who underwent on-pump coronary artery bypass grafting surgery were excluded from the analysis. All patients who presented with symptoms such as chest pain (angina pectoris) lasting for 20 min or longer despite the use of nitroglycerin were operated on within the first 24 h after admission to our hospital.

We used the left internal mammary artery (LIMA), right internal mammary artery (RIMA), radial artery, great saphenous vein and/or small saphenous vein for future anastomosis, based on previous risk factors. Intravenous heparin was administered in all cases to achieve an activated clotting time of more than 300 s. An automatic pod spreader was used for a better visualization of the anastomotic site. Afterwards, the coronary arteries were longitudinal incised. The coronary arteries were then longitudinally incised, and temporary shunts were placed into the lumen of the targeted arteries to allow for continuous blood flow during anastomosis and to decrease the possibility of bleeding. Anastomosis was performed using 7-0 and 8-0 monofilament sutures. The decision to use temporary epicardial pacing was made by each surgeon. Standard anticoagulation protocols were used by all patients after the OPCAB procedure, which were similar in Cologne and Tuebingen. Our methods were previously described [22].

### 2.2. Data Collection

The data were withdrawn from the institutional database of the University hospital Cologne and the University hospital Tuebingen. All information was collected during patients’ hospital stay and analyzed retrospectively.

### 2.3. Outcome Analysis

All-cause in-hospital mortality after OPCAB surgery was the primary endpoint of our study. The secondary outcomes analyzed included dialysis, transient ischemic attack (TIA) with symptom duration less than 60 min, low cardiac output syndrome (LCOS), reoperation due to postoperative bleeding, wound infection, and duration of hospital stay.

### 2.4. Ethics

This research was conducted in accordance with the Declaration of Helsinki (revised version of 2013). The Ethics Committee of the Medical Faculty of the University of Cologne and the Ethics Committee of the Medical Faculty of the University of Tuebingen confirmed that under German law, the authors did not require a separate ethical approval. All purely retrospective clinical studies can be conducted without an ethical statement.

### 2.5. Statistical Methods

Statistical analysis was conducted using Student’s *t*-test or Mann–Whitney-U test, depending on whether continuous variables were normally distributed or not. The Chi-square test was used for categorical variables. Normally distributed samples were presented as the mean ± standard deviation (SD). Fisher’s exact test was used when the minimum expected count of cells was less than 5. The optimal cut-off values were defined as values that provided highest sensitivity and specificity. Logistic regression was used to create the predicted variable. The PSM analysis was applied to even groups and provide statistical comparison. A *p*-value of less than 0.05 was considered significant. Statistical analysis was performed using Statistical Package for Social Sciences, version 28.1 (SPSS Inc., Chicago, IL, USA).

## 3. Results

### 3.1. Preoperative Data before and after PSM

Preoperative characteristics of the two groups (prior to PSM) are presented in Table 1 for men (n = 255) and woman (n = 88). Following PSM, the groups were reduced to 61 men and 61 women. The mean age was 66 ± 9 years for men and 71 ± 8 years for women. The majority of patients were classified as overweight or obese, with mean BMIs of 29.3 ± 9.7 and 30.2 ± 5.1 kg/m^2^ for males and females. After 1:1 propensity score matching, the groups were well balanced. No significant differences were observed between the male and female groups in terms of age (*p* = 0.116), BMI (*p* = 0.221), diabetes (*p* = 0.853), cardiogenic shock (0.256), STEMI (*p* = 0.283), NSTEMI (*p* = 0.555), or dialysis (*p* = 0.496).

### 3.2. Intraoperative Characteristics before and after PSM

Intraoperative data for both groups before (men, n = 255; women, n = 88) and after PSM (men, n = 61; women, n = 61) are presented in Table 2. The use of both internal thoracic arteries (ITA) grafts was significantly higher (*p* = 0.043) in the male group compared to the female group before PSM. However, after PSM, the use of both ITA grafts was similar in both groups (*p* = 0.412). Additionally, male patients underwent total-arterial revascularization with the T-graft technique significantly more frequently than female patients before PSM (*p* < 0.001) and after PSM (*p* = 0.005). The mean operation time was significantly longer (*p* = 0.001) in the male group than in the female group. Other data did not differ significantly between the two groups.

### 3.3. Primary and Secondary Outcomes

Primary and secondary outcomes for both groups before (men, n = 255; women, n = 88) and after PSM (men, n = 61; women, n = 61) are summarized in Table 3. The in-hospital mortality rate was not significantly higher (*p* = 0.496) in the female group compared to the male group. Furthermore, there was no significant difference in the mean length of ICU (*p* = 0.529) or hospital stay (*p* = 0.930) between both groups. Likewise, in the secondary outcomes (transient ischemic attack (*p* = 0.365), low cardiac output syndrome after surgery (*p* = 0.644), dialysis (*p* = 0.496), reoperation due to postoperative bleeding (*p* = 0.691), and wound infection rate (*p* = 0.187)), no significant differences were observed between the unadjusted and adjusted groups.

## 4. Discussion

We investigated gender-related discrepancies in short-term outcomes in patients who underwent OPCAB surgery only. Our analysis showed that all-cause in-hospital mortality rate was not significantly higher (*p* = 0.496) in the female group compared to the male group. Likewise, in the secondary outcomes (transient ischemic attack (*p* = 0.365), low cardiac output syndrome after off-pump surgery (*p* = 0.644), dialysis (*p* = 0.496), reoperation due to postoperative bleeding (*p* = 0.691), wound infection rate (*p* = 0.187), and duration of hospital stay (*p* = 0.930)), no significant differences were observed between the two above-mentioned groups.

Studies on gender differences after OPCAB surgery are scarce [16,17,18,19,20]. Previous retrospective trials have shown that female gender might be an independent risk factor for operative mortality [14,23]. Similarly, Alam et al. [24] found increased operative and 30-day mortality rates in females compared to males after bypass surgery. However, the authors did not analyze the potential impact of the operative technique on patient’s survival, which could affect results and lead to potential bias [24]. In general, females present more often relevant comorbidities such as diabetes mellitus, hyperlipidemia, arterial hypertension and peripheral vascular disease compared to males [8,25]. In addition, women suffered more commonly from chronic renal insufficiency [14]. All of the above-mentioned factors could lead to poor outcomes during the postoperative period [14,25]. In contrast, we could not find any statistically significant differences regarding preoperative comorbidities between both groups in our study.

Generally, women undergo less frequent and significantly later coronary revascularization due to protective hormone levels in the premenopausal period [26]. These factors could explain the higher mortality rate by women compared to men by cardiogenic shock after CABG procedure [27]. In contrast, Amato et al. [28] stated that female gender was not an independent predictor for higher mortality after CABG surgery. Ennker et al. [29] analyzed 12,606 patients who underwent bypass surgery in their study. After adjusting for preoperative risk factors, the authors could not find any significant difference in the mortality rate between male and female groups [29]. Likewise, the mortality rate was not statistically significantly different (*p* = 0.496) in our analysis.

Moreover, further authors showed that the operative technique had no impact on in-hospital mortality after OPCAB surgery [30]. However, difficulties with anastomoses due to a smaller size of the coronary artery by women could affect results [31]. The increased risk of thrombosis, especially near the suture line, should be taken into consideration [30,31]. The use of multiple arterial grafts to compensate for these risk factors remains controversial [11,30,31]. Rocha et al. [31] showed improved outcomes in females despite a significantly lower use of arterial grafts compared to males. Moreover, Kurlansky et al. [32] showed no difference in survival after 12 years using propensity-matched analysis of the female group undergoing cardiac surgery with multiple artery grafting versus single artery grafting. The authors suggested that multiple artery grafting surgery might benefit results by younger patients [32,33]. Likewise, in our study, the use of arterial grafts was significantly higher (*p* = 0.005) in the male group compared to the female group. Despite all of the above-mentioned factors, we were unable to identify a higher survival rate in the male group compared to the female group in our sample.

Based on the information provided, the use of OPCAB technique to improve outcomes has been controversially discussed in recent years [9,14,21]. Puskas et al. [21] found a decreased rate of major adverse events among female patients undergoing OPCAB surgery compared to on-pump CABG procedure. Others, such as one by Fu et al. [9] showed an increased risk of major cardiac and cerebral events by female patients after the OPCAB procedure. The authors mentioned that incomplete revascularization due to the smaller intraluminar diameter of coronary arteries could explain an increased rate of late adverse events in females [9]. However, the survival rate was similar in both gender groups [9,21]. Similarly, in our sample, we did not find any benefits of the OPCAB procedure with regard to morbidity and mortality between both sexes.

Moreover, several studies stated that female patients suffered more often from postoperative complications after bypass surgery [9,34]. The authors stated that female sex could be a significant risk factor for acute renal failure [9,34]. Chou et al. [34] showed that women underwent dialysis significantly earlier compared to men after bypass procedure. In contrast, the observational animal studies showed that male sex might be associated with an increased incidence of acute kidney injury requiring dialysis [35,36]. Moreover, Neugarten et al. [36] highlighted the protective role of female sex in the development of acute renal failure in patients after open-heart surgery. The authors mentioned that this protective effect could be explained by effects of sex hormones on cellular processes in the pathogenesis of acute renal failure [35,36]. In contrast, we could not identify any significant differences regarding acute kidney failure (*p* = 0.956) and dialysis (*p* = 0.456) between the male and female group in our study.

Female gender was associated with a higher prevalence of wound infection after bypass surgery compared to male gender [37]. Additionally, the authors reported that female gender remained a strong predictor of wound infection after the CABG procedure [38]. Furthermore, Patel et al. [20] observed that females had a significantly higher incidence of wound infection (*p* = 0.028) compared to males after the off-pump procedure only. However, we found no significant differences (*p* = 0.187) in wound infection between both genders after OPCAB surgery.

No significant differences in primary and secondary outcomes were observed between men and women after OPCAB surgery. Therefore, further prospective trials with larger sample sizes are needed to identify any potential sex differences in patients after off-pump bypass procedure.

## 5. Study Limitations

This purely retrospective clinical research has several limitations. Firstly, it is a retrospective double-center analysis with a relatively small patient cohort. Secondly, we focused on short-term outcomes and did not pay enough attention to the long-term results. Thirdly, specific pathophysiological conditions were not evaluated in our study. Fourthly, OPCAB surgery was conducted by different surgeons, which may have introduced bias in our findings. All potential biases and confounders should be taken into account in further studies. Lastly, the sample size was not calculated, which could potentially result in lower statistical power.

## 6. Conclusions

Based on our findings, gender did not have a significant impact on short-term outcomes following OPCAB surgery. Mortality rates were similar in both male and female groups, and secondary outcomes did not differ significantly between the two groups. As a result, OPCAB surgery appears to be a safe procedure for both male and female patients.

## Figures and Tables

**Table 1:** Preoperative data.

	Before PSM			After PSM		
	Male (n = 255)	Female (n = 88)	*p*-Value	Male (n = 61)	Female (n = 61)	*p*-Value
Age (years), mean ± SD	66 ± 9	71 ± 8	0.237	68 ± 9	72 ± 8	0.116
BMI (kg/m^2^), mean ± SD	29.3 ± 9.7	30.2 ± 5.1	0.426	29.7 ± 17.4	30.4 ± 4.5	0.751
Euroscore II (%), mean ± SD	3.9 ± 2.3	4.9 ± 2.5	0.093	3.9 ± 2.0	5.1 ± 2.4	0.135
LV-EF (%), mean ± SD	48 ± 13	46 ± 13	0.274	46 ± 14	47 ± 15	0.830
Left main coronary artery disease, n (%)	107 (42.8%)	32 (36.8%)	0.326	29 (48.3%)	20 (33.3%)	0.095
NSTEMI, n (%)	86 (33.9%)	27 (30.7%)	0.585	20 (32.8%)	17 (27.9%)	0.555
STEMI, n (%)	35 (13.8%)	15 (17.0%)	0.463	6 (9.8%)	10 (16.4%)	0.283
Cardiogenic shock, n (%)	31 (12.2%)	10 (11.4%)	0.834	9 (14.8%)	5 (8.2%)	0.256
Previous stenting, n (%)	83 (32.8%)	20 (22.7%)	0.076	22 (36.1%)	13 (21.3%)	0.072
Previous stroke, n (%)	13 (5.1%)	6 (6.8%)	0.591	4 (6.6%)	6 (9.8%)	0.509
Reoperation, n (%)	23 (9.1%)	5 (5.9%)	0.353	3 (4.9%)	3 (4.9%)	0.660
Diabetes, n (%)	99 (38.8%)	39 (44.3%)	0.365	24 (39.3%)	25 (41.0%)	0.853
HbA1C (%), mean ± SD	6.5 ± 1.5	6.3 ± 0.9	0.298	6.6 ± 1.9	6.4 ± 0.9	0.429
PVD, n (%)	58 (22.9%)	17 (19.5%)	0.511	15 (24.6%)	11 (18.3%)	0.402
Arterial hypertension, n (%)	241 (94.5%)	86 (97.7%)	0.377	59 (96.7%)	61 (100%)	0.496
Pulmonary hypertension, n (%)	7 (2.8%)	6 (6.8%)	0.106	0 (0.0%)	3 (4.9%)	0.244
Hyperlipidemia	227 (89.0%)	81 (92.0%)	0.419	53 (86.9%)	57 (93.4%)	0.224
Deep vein thrombosis, n (%)	1 (0.4%)	1 (1.1%)	0.450	0 (0.0%)	1 (1.6%)	0.315
Pulmonary embolism, n (%)	4 (1.6%)	1 (1.1%)	0.765	1 (1.6%)	1 (1.6%)	1.000
Obstructive sleep apnea, n (%)	26 (10.2%)	4 (4.5%)	0.104	7 (11.5%)	2 (3.3%)	0.163
COPD, n (%)	38 (15.0%)	15 (17.0%)	0.651	9 (14.8%)	10 (16.4%)	0.803
Dialysis, n (%)	8 (3.2%)	3 (3.4%)	0.910	0 (0.0%)	2 (3.3%)	0.496
Chronic renal insufficiency, n (%)	33 (13.0%)	8 (9.1%)	0.326	9 (14.8%)	7 (11.5%)	0.592
CK, U/L, mean ± SD	157 ± 200	140 ± 164	0.902	175 ± 271	129 ± 150	0.214
CK-MB, U/L, mean ± SD	28 ± 33	31 ± 29	0.924	25 ± 26	31 ± 33	0.223
Lactate 48 h, mean ± SD	1.0 ± 0.4	0.9 ± 0.4	0.739	1.0 ± 0.4	1.0 ± 0.5	0.989
Creatinine 48 h, mg/dL, mean ± SD	1.3 ± 2.9	0.9 ± 0.3	0.182	1.0 ± 0.3	0.9 ± 0.3	0.278

LV-EF, left ventricular ejection fraction; PVD, peripheral vascular disease; BMI, body mass index; COPD, chronic obstructive pulmonary disease; STEMI, ST-elevation myocardial infarction; NSTEMI, non-ST-elevation myocardial infarction; PSM, propensity score matching; CK, creatine kinase; CK-MB, creatine kinase MB.

**Table 2:** Intraoperative data.

	Before PSM			After PSM		
	Male (n = 255)	Female (n = 88)	*p*-Value	Male (n = 61)	Female (n = 61)	*p*-Value
Use of 2 ITA grafts, n (%)	142 (56.8%)	39 (44.3%)	0.043	33 (55.0%)	29 (47.5%)	0.412
“Totally arterial” revascularization, n (%)	129 (51.6%)	34 (39.5%)	0.005	38 (63.3%)	28 (45.9%)	0.005
T-graft technique, n (%)	131 (52.4%)	27 (31.4%)	<0.001	34 (56.7%)	19 (31.1%)	0.005
Heartstring, n (%)	87 (35.1%)	40 (45.5%)	0.085	17 (28.8%)	26 (42.6%)	0.115
Temporary pacer, n (%)	142 (57.0%)	53 (60.2%)	0.601	29 (48.3%)	42 (68.9%)	0.022
IABP intraoperative, n (%)	7 (2.8%)	0 (0.0%)	0.198	1 (1.7%)	0 (0.0%)	0.315
ECMO intraoperative, n (%)	2 (0.8%)	0 (0.0%)	0.404	0 (0.0%)	0 (0.0%)	
Catecholamine use, n (%)	243 (97.6%)	87 (98.9%)	0.644	56 (98.3%)	61 (100%)	0.337
Operation time, min, mean ± SD	179 ± 39	158 ± 28	0.008	184 ± 28	157 ± 30	0.001

IABP, intra-aortic balloon pump; ITA, internal thoracic artery; SVG, saphenous vein grafts; PSM, propensity score matching; ECMO, extracorporeal membrane oxygenation.

**Table 3:** Postoperative data.

	Before PSM			After PSM		
	Male (n = 163)	Female (n = 52)	*p*-Value	Male (n = 52)	Female (n = 52)	*p*-Value
TIA, n (%)	7 (2.8%)	6 (6.8%)	0.106	1 (1.7%)	4 (6.6%)	0.365
Stroke, n (%)	1 (0.4%)	0 (0.0%)	0.555	0 (0.0%)	0 (0.0%)	
Postoperative delirium, n (%)	31 (12.3%)	10 (11.4%)	0.825	8 (13.3%)	7 (11.5%)	0.757
ALS with ROSC, n (%)	9 (3.5%)	1 (1.1%)	0.463	2 (3.3%)	0 (0.0%)	0.154
Low cardiac output syndrome after surgery, n (%)	9 (3.6%)	3 (3.4%)	0.934	3 (4.9%)	3 (4.9%)	0.644
CK, 48 h, U/L, mean ± SD	798 ± 1088	531 ± 783	0.065	639 ± 815	522 ± 882	0.519
CK-MB, 48 h, U/L, mean ± SD	34 ± 117	29 ± 50	0.363	23 ± 14	28 ± 57	0.541
Lactate 48 h, mmol/L, mean ± SD	1.5 ± 1.0	1.6 ± 1.4	0.531	1.3 ± 0.4	1.7 ± 1.7	0.230
Creatinine 48 h, mg/dL, mean ± SD	1.2 ± 2.1	1.0 ± 0.5	0.452	1.0 ± 0.3	1.1 ± 0.6	0.190
Acute kidney failure, n (%)	20 (7.9%)	5 (5.7%)	0.485	5 (8.5%)	5 (8.2%)	0.956
Dialysis, n (%)	13 (5.1%)	2 (2.3%)	0.371	0 (0.0%)	2 (3.3%)	0.496
Wound infection, n (%)	26 (10.2%)	6 (6.8%)	0.343	7 (11.5%)	3 (4.9%)	0.187
Plastic covering of the wound, n (%)	4 (1.6%)	1 (1.1%)	0.768	0 (0.0%)	0 (0.0%)	
Heart block requiring pacemaker implantation, n (%)	6 (2.4%)	0 (0.0%)	0.346	2 (3.4%)	0 (0.0%)	0.240
Bleeding requiring reoperation, n (%)	10 (3.9%)	3 (3.4%)	0.823	2 (3.3%)	2 (3.3%)	1.000
ICU stay, days, mean ± SD	2 ± 3	2 ± 4	0.583	2 ± 3	2 ± 4	0.870
Hospital stay, days, mean ± SD	9 ± 7	10 ± 6	0.844	9 ± 7	9 ± 6	0.657
Mortality rate (in-hospital), n (%)	5 (2.0%)	3 (3.4%)	0.429	0 (0.0%)	2 (3.3%)	0.496

TIA, transient ischemic attack; ICU, intensive care unit; CK-MB, creatine kinase-MB; LV-EF, left ventricular ejection fraction; ALS, advanced life support, PSM, propensity score matching, CK, creatine kinase; CK-MB, creatine kinase MB.

## Data Availability

The data are available on reasonable request.
